# On the Operation for the Aneurism

**Published:** 1800-06

**Authors:** Henry Reeve

**Affiliations:** Norwich


### *On the Operation for the Aneuriſm.*

——— "Laudatur ab his, culpatur ab illis."
HORAT. Sat. II. Lib. 1.

THE various improvements that have taken place in the operations of ſurgery, within theſe laſt forty years, are certainly ſufficient to attract the attention, and claim the acknowledgement, of all profeſſional men. Yet, upon enquiry, perhaps, we ſhall find, that we have not made ſuch conſiderable advances advances to perfection as might have been expected from our additional knowledge of anatomy and physiology; nor, though assisted by the accumulated experience of the ancients, have we yet been able to diminish, or totally to remove, what have too long been styled the "opprobria medicorum." Among those subjects that still remain to be improved and established by our increasing knowledge and discoveries, there is no one of greater importance to us as men, or more worthy our attention as surgeons, than the treatment of the Aneurism.

If we look back into the history of our art, we shall find the writers on surgery, fully acquainted with the nature and importance of the disease, explaining the different causes and situations of it; and also describing the method of operating for the cure, in particular parts of the body. And it does not appear that our knowledge of the disease is increased, or that we have arrived at a much greater degree of certainty in the treatment of it, since the days of Ætius and Paulus Ægineta.

It is true, the component parts of the human body have lately been more minutely examined and explained by the discoveries in chemistry; the circulation of the blood through the inosculating arteries has been more clearly ascertained and better understood; the doctrine of absorption has been more thoroughly canvassed and more accurately defined: but how far these theories are compatible with practice, and are likely to be attended with permanent advantage to the "ars medendi," it still remains for future experience and observation to determine. In aneurisms in the arm, we undertake the cure with more confidence and certainty of success; and from the respectable testimony of Heister, Dr. Monro, and Dr. Wm. Hunter, nobody would hesitate to recommend the performance of the operation. The reasons that encourage us to hope for success in the superior extremities, are plain and obvious; the arteries coming more immediately from the aorta, the ramifications being more numerous, and the circulation in all the vessels more strong and vigorous. It may here be remarked, that an improvement in the operation for the popliteal aneurism, was suggested by the justly celebrated John Hunter, viz. the taking up the femoral artery on the anterior part of the thigh, without doing any thing to the tumour in the ham; and this ingenious proposal has been sanctioned by experience. The numerous successful cases upon record seem sufficient to justify the attempt to save the limb, by including the vessel in a ligature in the popliteal aneurism, and even in some cases where the disease is situated in the femoral artery; but, when the aneurism is high up the thigh, or in the groin, which sometimes occurs, the young practitioner may be frequently at a loss loſs what conduct to purſue. He may be perplexed or divided in his own mind, by the various opinions of different authors; he finds the operation of taking up the diſeaſed artery with a ligature, ſtrenuouſly recommended, and as vehemently condemned. On the one hand, he is encouraged with the moſt animating proſpects of ſucceſs, by the plauſible theory of the ingenious John Bell; on the other hand, he is told by the learned experience and profound judgment of Pott, that the operation had never, within *his* obſervation, been ſucceſsful, and, in his opinion, ought never to be attempted.— The ingenuity of Mr. Bell's reaſoning, and the ſlender teſtimony of Guattani, cannot ſurely determine the matter, when ſuch an eminent ſurgeon, and ſo many reſpectable practitioners of the preſent day, entertain the very oppoſite ſentiments. What has been ſaid upon the treatment of aneuriſms, in a modern ſyſtem of ſurgery, does not appear ſufficient to direct the unexperienced judgment of a tyro in the profeſſion, nor ſufficient to enable him, with confidence, to decide what plan to adopt for the cure. It is to be regretted, therefore, that a collection of *facts* has not been publiſhed, and a comparative view taken of the average of ſucceſsful caſes where the operation has been performed; by which means every one might be enabled, in ſome meaſure, to form his own opinion, and act from his own judgment, without being directed by the fallacious teſtimony of a ſingle caſe, or biaſſed by the prejudiced voice of an individual.

